# Therapeutic potential of fibrinogen γ-chain peptide-coated, ADP-encapsulated liposomes as a haemostatic adjuvant for post-cardiopulmonary bypass coagulopathy

**DOI:** 10.1038/s41598-020-68307-5

**Published:** 2020-07-09

**Authors:** Osamu Ishida, Kohsuke Hagisawa, Nozomu Yamanaka, Koji Tsutsumi, Hidenori Suzuki, Masato Takikawa, Shinji Takeoka, Manabu Kinoshita

**Affiliations:** 1 Department of Cardiovascular Surgery, National Defense Medical College, 3-2 Namiki, Tokorozawa-shi, Saitama 359-8513 Japan; 2 Department of Physiology, National Defense Medical College, Tokorozawa, Japan; 3 Division of Morphological and Biomolecular Research, Graduate School of Medicine, Nippon Medical School, Tokyo, Japan; 4 Department of Advanced Science and Engineering, Graduate School of Advanced Science and Engineering, Waseda University, Tokyo, Japan; 5 Department of Life Science and Medical Bioscience, Graduate School of Advanced Science and Engineering, Waseda University, Tokyo, Japan; 6 Department of Immunology and Microbiology, National Defense Medical College, Tokorozawa, Japan

**Keywords:** Preclinical research, Translational research, Platelets

## Abstract

Fibrinogen γ-chain peptide-coated, adenosine 5′-diphosphate (ADP)-encapsulated liposomes (H12-ADP-liposomes) are a potent haemostatic adjuvant to promote platelet thrombi. These liposomes are lipid particles coated with specific binding sites for platelet GPIIb/IIIa and encapsulating ADP. They work at bleeding sites, facilitating haemostasis by promoting aggregation of activated platelets and releasing ADP to strongly activate platelets. In this study, we investigated the therapeutic potential of H12-ADP-liposomes on post-cardiopulmonary bypass (CPB) coagulopathy in a preclinical setting. We created a post-CPB coagulopathy model using male New Zealand White rabbits (body weight, 3 kg). One hour after CPB, subject rabbits were intravenously administered H12-ADP-liposomes with platelet-rich plasma (PRP) collected from donor rabbits (H12-ADP-liposome/PRP group, n = 8) or PRP alone (PRP group, n = 8). Ear bleeding time was greatly reduced for the H12-ADP-liposome/PRP group (263 ± 111 s) compared with the PRP group (441 ± 108 s, p < 0.001). Electron microscopy showed platelet thrombus containing liposomes at the bleeding site in the H12-ADP-liposome/PRP group. However, such liposome-involved platelet thrombi were not observed in the end organs after H12-ADP-liposome administration. These findings suggest that H12-ADP-liposomes could help effectively and safely consolidate platelet haemostasis in post-CPB coagulopathy and may have potential for reducing bleeding complications after cardiovascular surgery with CPB.

## Introduction

Platelet transfusions are broadly and commonly used for the prevention or treatment of bleeding in settings such as haematologic disorder, trauma, and surgery^1,2^. Globally, the supply of blood products is currently insufficient to meet demand, although this is influenced in part by the socio-economic background of the countries^3–5^. In ageing society where the blood-donating population is decreasing and the blood-consuming population is increasing, blood shortages are anticipated to become chronic and critical^4,6^. For example, in Japan, as long as current blood donation behaviour continues, the estimated shortfall of blood donations is expected to double from 2025 to 2050^7^. In addition, it is extrapolated that the scarcity of platelet preparations will certainly increase in the future because of their limited shelf life and storage methods^8^. Therefore, effective countermeasures against the lack of platelet concentrates should be prepared. Alternatives to platelet transfusion have long been a subject of intense investigation^2,9^. Platelet-derived haemostatic products, such as lyophilised platelets, platelet-derived microparticles, and infusible platelet membranes are not in clinical use^9^. Platelets derived from stem cells are under active investigation and appear very promising^10,11^, but this technology is not yet ready for clinical application. Synthetic platelet substitutes have the advantage of ameliorating the limitations inherent to natural platelet-derived products^9,12^. Several types of synthetic platelet substitute have been proposed, characterised by enhanced platelet aggregation and a reduced need for the use and storage of platelet concentrates^9,12^.

Fibrinogen γ-chain (dodecapeptide HHLGGAKQAGDV [H12])-coated, adenosine 5′-diphosphate-encapsulated liposomes (H12-ADP-liposomes) are a potent haemostatic adjuvant to promote platelet thrombi. H12-ADP-liposomes are lipid particles coated with a specific binding site for platelet GPIIb/IIIa (mean diameter of 150 nm). They bind to activated platelets using the H12-GPIIb/IIIa interaction and release ADP^13,14^. According to pharmacokinetic studies^15^, H12-ADP-liposomes are intact in the blood circulation for 24 h, distributed mainly in the liver and spleen, and are biodegraded and eliminated from the body within 7 days after injection. The haemostatic ability of H12-ADP-liposomes has been reported in rats and rabbits with busulfan-induced thrombocytopaenia^13^ and rabbit models of thrombocytopaenic massive haemorrhage from acute liver injury^16–18^.

Post-cardiopulmonary bypass (CPB) coagulopathy is a complex entity involving several factors, including platelet dysfunction, consumptive coagulopathy, and systemic inflammation response^19^. Because post-CPB coagulopathy underlies massive bleeding after cardiovascular surgery using CPB^20^, medical countermeasures against this complication are important to reduce postoperative bleeding complications. Antifibrinolytics are the only ones recommended in the guidelines^21,22^ and routinely used in clinical practice^23^, but their limited effectiveness leaves room for further research and development of novel agents. Thus, this study aimed to assess the feasibility of H12-ADP-liposomes in a rabbit CPB model and to evaluate the efficacy of H12-ADP-liposome on coagulopathy caused by CPB.

## Results

### Characteristics of H12-ADP-liposome

The size and the zeta potential of H12-ADP-liposomes were 153 ± 42 nm and − 10.3 ± 1.0 mV, respectively (supplement). When the total lipid concentration was 20 mg/mL, the concentrations of ADP inside and outside of liposomes were 0.066 and 0.001 mg/mL, respectively, and the encapsulation efficiency was 9.3%. The concentration of H12-PEG-Glu2C18 was 0.64 mg/mL, which is equivalent to 0.37 mol% of the liposome composition. Besides, the concentration of endotoxin was under the detection limit.

### Rabbit model of post-CPB coagulopathy

**Figure 1:**
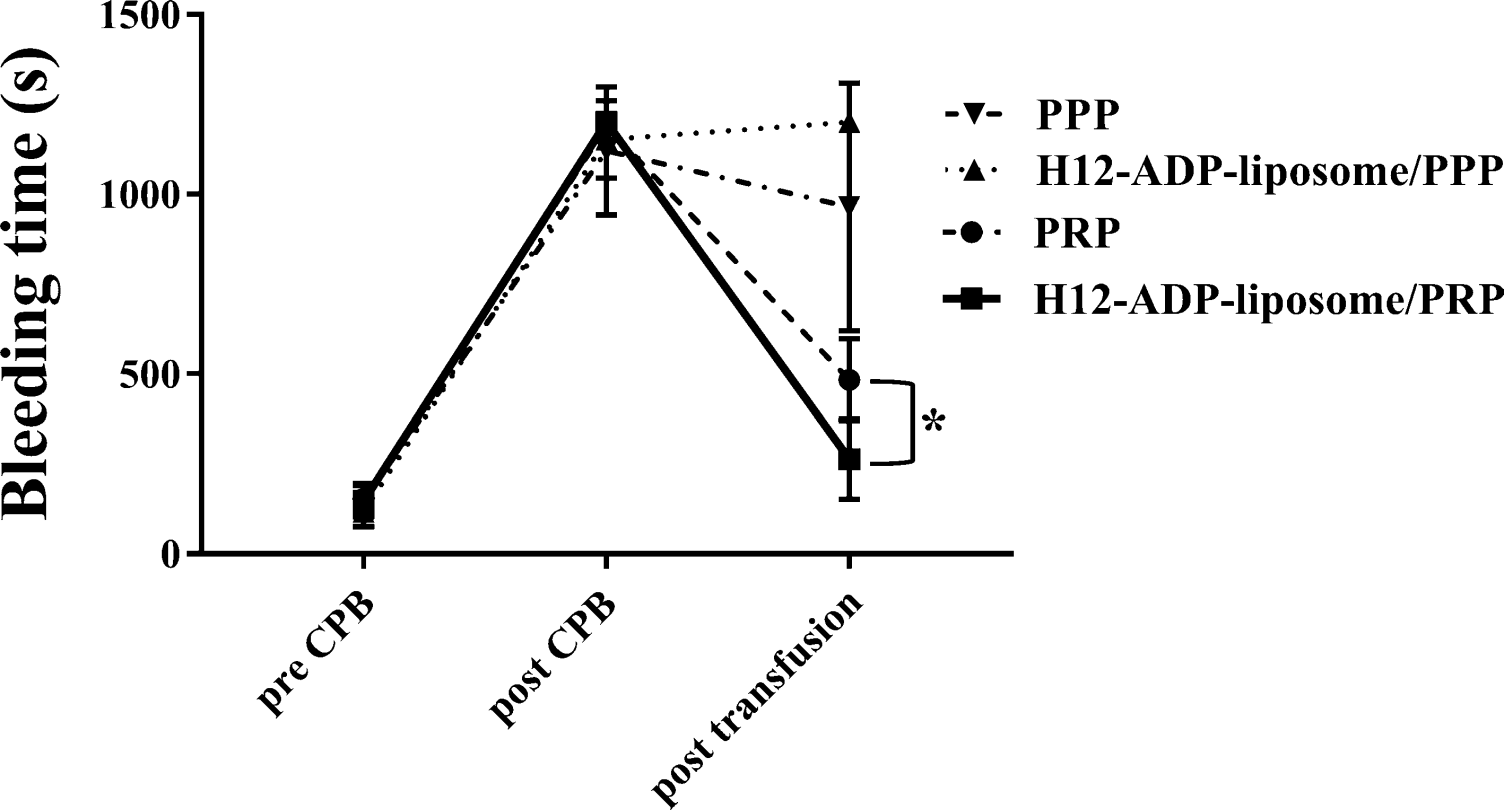
Change in ear bleeding time measured to evaluate overall haemostatic capacity. Note that H12-ADP-liposome/PRP group significantly improved/reduced the prolonged ear bleeding time than the PRP group. Data are means ± standard deviation from five mice in PPP and H12-ADP-liposome/PPP groups and eight mice in PRP and H12-ADP-liposome/PRP groups. *p < 0.001. PPP, platelet-poor plasma; PRP, platelet-rich plasma.

**Figure 2:**
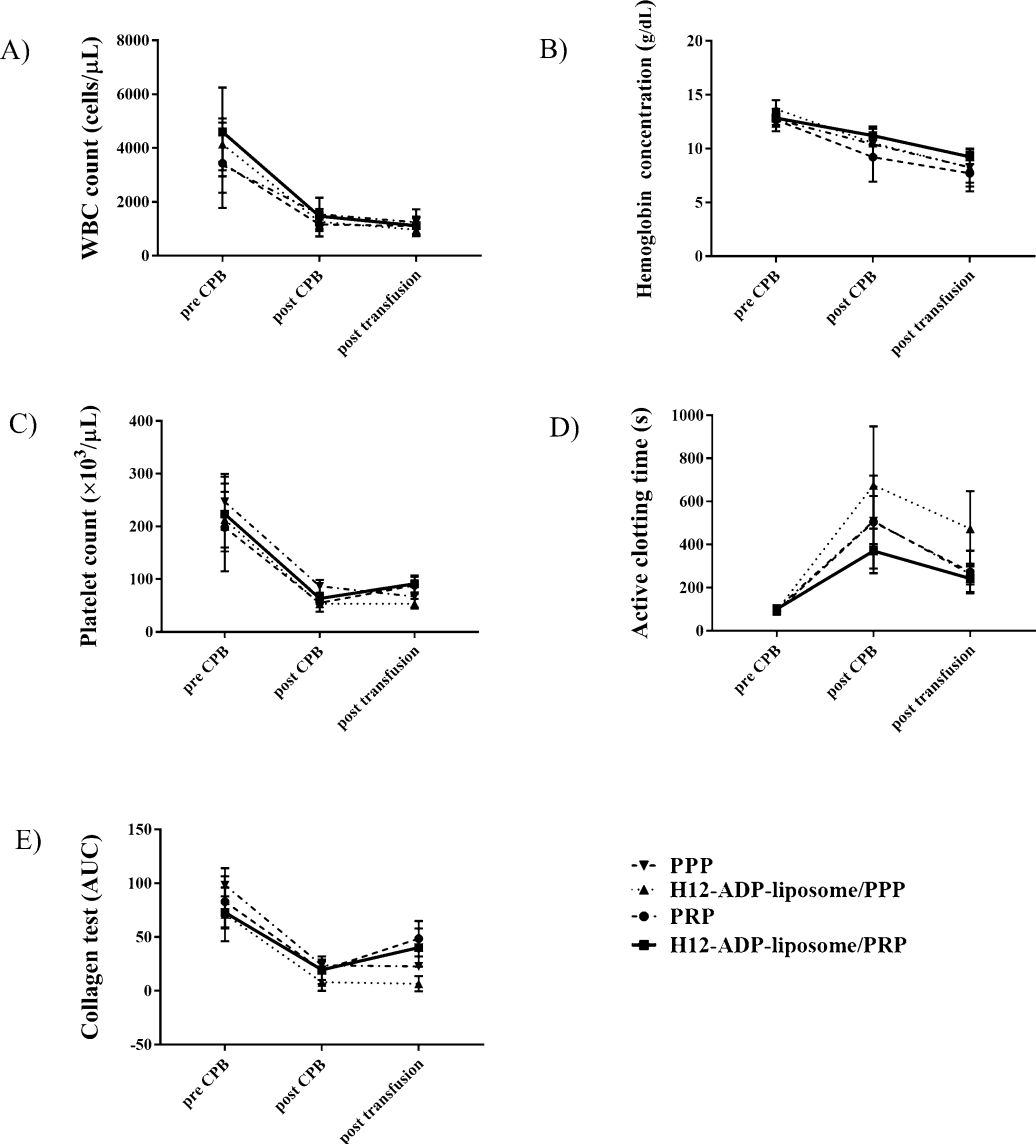
Changes in CBC, coagulation function, and platelet function. (**A**) White blood cell (WBC) count (cells/μL). (**B**) Haemoglobin (Hb) concentration (g/dL). (**C**) Platelet count (× 10^3^ cells/μL). (**D**) Coagulation function test using the Sonoclot coagulation analyser recorded as activated clotting time (s). (**E**) Platelet function test using the Multiplate analyser assessed as the collagen test (AUC). Data represent mean ± standard deviation from five mice in the PPP and H12-ADP-liposome/PPP groups and from eight mice in PRP and H12-ADP-liposome/PRP groups. PPP, platelet-poor plasma; PRP, platelet-rich plasma.

**Figure 3:**
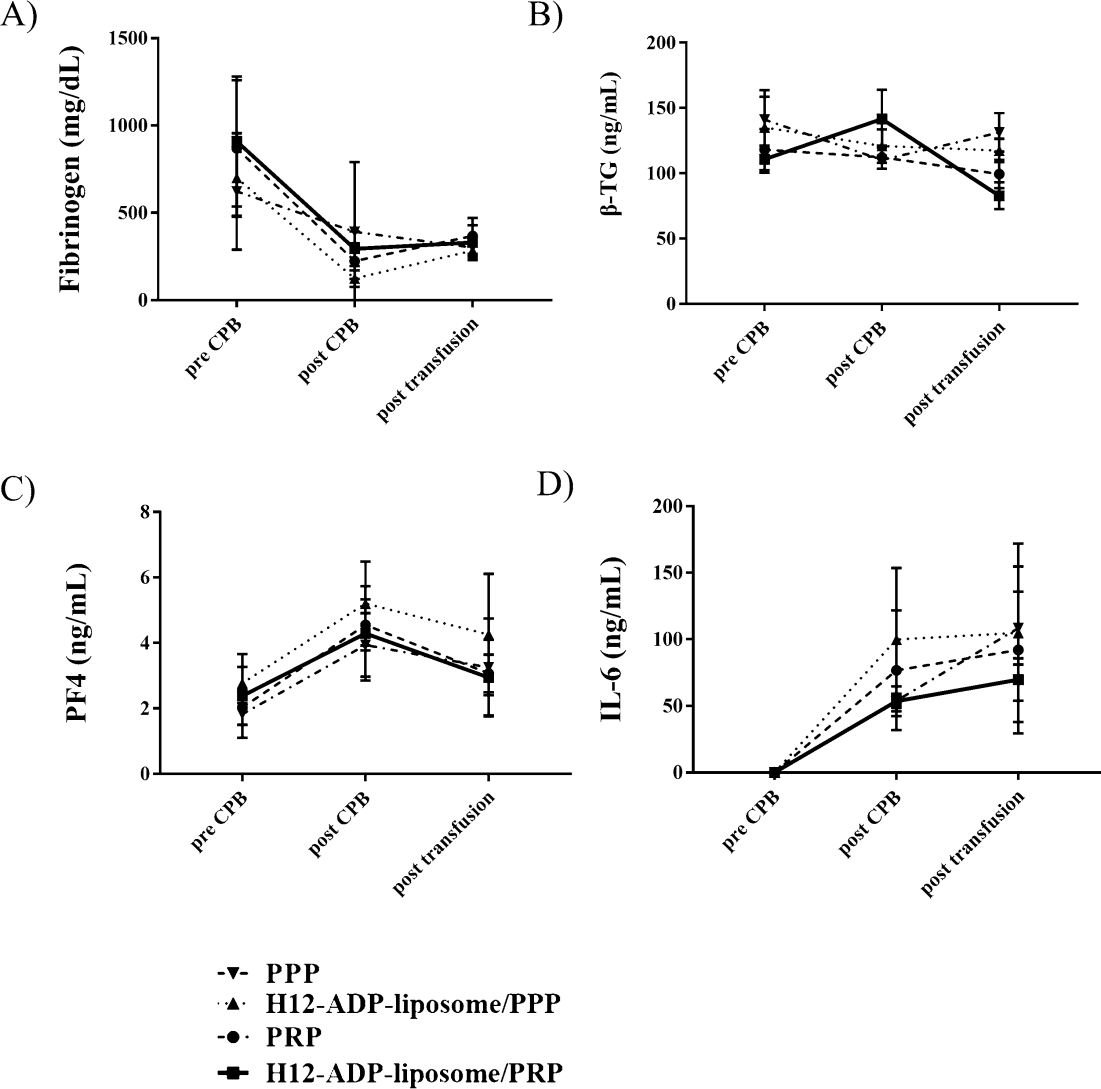
Results of ELISA employed to assess coagulation function, platelet activation, and systemic inflammation. (**A**) Fibrinogen concentration (mg/dL). (**B**) Concentration of β-thromboglobulin (β-TG) (ng/mL). (**C**) Concentration of platelet factor 4 (PF4) (ng/mL). (**D**) Concentration of IL-6 (ng/mL). Data represent mean ± standard deviation from five mice in the PPP and H12-ADP-liposome/PPP groups and eight mice in the PRP and H12-ADP-liposome/PRP groups.

We created a rabbit model of CPB, and the validity of this model can be examined by comparing measurements before and after CPB. Bleeding time, as one of the most important indicators of postoperative bleeding complications, was drastically prolonged after 1 h of CPB (Fig. 1, Table 1). Platelet counts were also severely decreased after CPB (Fig. 2C, Table 1), indicating that the current CPB protocol induced coagulopathy in rabbits. Activated clotting time (ACT) was consistently prolonged, but collagen test and fibrinogen concentration were decreased after CPB (Figs. 2D,E, 3A). White blood cell (WBC) was steadily decreased because of haemodilution with CPB, and haemoglobin (Hb) concentration was slightly decreased, probably due to haemolysis by CPB (Fig. 2A,B). Plasma β-thromboglobulin (β-TG) levels were not significantly altered after CPB (Fig. 3B). Plasma platelet factor 4 (PF4) and interleukin (IL)-6 levels were increased after CPB, which depicted platelet activation and inflammation caused by CPB (Fig. 3C,D).

**Table 1:** Results of the evaluation for complete blood count, coagulation function, platelet function.

	PPP			PPP + H12			PRP			PRP + H12		
	preCPB	postCPB	post T/F	preCPB	postCPB	post T/F	preCPB	postCPB	post T/F	preCPB	postCPB	post T/F
**Blood cell count**												
WBC (cells/ )	3,360 ± 1,415	1,540 ± 619	1,240 ± 483	3,767 ± 1,150	1,167 ± 231	625 ± 153	3,511 ± 1,046	1,167 ± 418	1,077 ± 338	4,600 ± 1648	1,471 ± 288	1,114 ± 343
Hb (g/dL)	12.7 ± 0.7	10.4 ± 1.1	8.3 ± 1.4	13.3 ± 0.9	10.6 ± 0.4	8.1 ± 1.5	12.5 ± 1.1	9.4 ± 2.2	7.8 ± 1.6	12.8 ± 0.6	11.2 ± 0.9	9.2 ± 0.7
Pit (× 10^3^/µL)	247 ± 53	86 ± 12	67 ± 20	236 ± 42	63 ± 13	58 ± 10	200 ± 78	58 ± 18	89 ± 15	224 ± 71	64 ± 13	91 ± 16
**Sonoclot**												
ACT (s)	100 ± 11	509 ± 117	263 ± 49	106 ± 15	729 ± 263	462 ± 206	89 ± 17	492 ± 205	264 ± 95	101 ± 17	370 ± 103	242 ± 63
**Multiplate**												
Collagen test (AUC)	98.2 ± 16.0	23.8 ± 4.8	22.8 ± 3.1	64.3 ± 31.5	7.3 ± 6.4	5.3 ± 4.7	84.8 ± 23.0	18.2 ± 12.3	45.0 ± 16.0	72.9 ± 15.0	19.6 ± 9.4	40.2 ± 17.6
Bleeding times	147 ± 43	1,120 ± 178	964 ± 345	128 ± 55	1,120 ± 139	1,200 ± 0	155 ± 40	1,070 ± 33	441 ± 108	145 ± 23	1,200 ± 0	263 ± 111

### Administration of H12-ADP-liposome potently augmented the platelet-rich plasma (PRP)-induced reduction in bleeding time after CPB

We focused on bleeding time to examine the effect of H12-ADP-liposomes on post-CPB coagulopathy, because this value has been seen to be markedly prolonged after CPB and is closely involved in postoperative bleeding from surgical sites. PPP treatment did not significantly reduce the bleeding time (Fig. 1). Thrombocytopaenia and platelet dysfunction may mainly cause post-CPB coagulopathy^19^, although decreases in coagulation factors such as fibrinogen have been observed after CPB (Fig. 3A). Additional treatment with H12-ADP-liposomes followed by platelet-poor plasma (PPP) treatment also did not ameliorate the prolonged bleeding time (Fig. 1). H12-ADP-liposomes do not aggregate by themselves, but instead promote or augment platelet aggregation^13^. Therefore, if platelet function is seriously damaged after CPB, H12-ADP-liposomes cannot effectively induce their haemostatic potential. A certain threshold level of normal functioning platelets is thus needed under such conditions.

As expected, platelet supplementation by PRP transfusion/treatment increased platelet counts after CPB to the same degree (PRP group, 89 ± 15 × 10^3^/μL; Fig. 2C), to over 50 × 10^3^/μL, but less than 100 × 10^3^/μL. Bleeding time was thus markedly reduced by PRP treatment (Fig. 1), but remained substantially higher than that before CPB (after PRP treatment, 441 ± 108 s; before CPB, 155 ± 40 s). Interestingly, additional treatment with H12-ADP-liposomes followed by PRP treatment strongly augmented the PRP-induced reduction of bleeding time (H12-ADP-liposome/PRP group, 263 ± 111 s; p < 0.001 compared with the PRP group), but showed almost the same platelet count as the PRP group (91 ± 16 × 10^3^/μL, Fig. 2C).

### Administration of H12-ADP-liposomes with PRP did not affect ACT or collagen test results

We examined the effects of additional treatment with H12-ADP-liposomes following PRP treatment on ACT and collagen test results after CPB. Although both H12-ADP-liposome/PRP and PRP groups shortened the prolonged ACT and increased the reduced area under the aggregation curve (AUC) seen with the collagen test, no difference in restoration of ACT or collagen test results was evident between the two groups (ACT: H12-ADP-liposome/PRP group, 242 ± 63; PRP group, 264 ± 95 s; collagen test AUC: 40 ± 18 vs. 45 ± 16, Fig. 2D,E). No significant changes in β-TG, PF4, or IL-6 levels were seen by transfusion of H12-ADP-liposome/PRP or PRP, and no differences in these parameters were seen between the two groups (Fig. 3, Table 2). These results may imply that administration of H12-ADP-lipsomes did not affect platelet activation and systemic inflammatory responses even in the PRP-transfused condition. H12-ADP-lipsomes may work only at the host bleeding sites where activated platelets (normal functioned) are present. These liposomes thus may not work within intact vascular spaces where the normal platelets are present (as no activated platelets are present).

**Table 2:** Results of the enzyme-linked immunosorbent assay to assess coagulation function, platelet activation, and systemic inflammation.

	PPP			PPP + H12			PRP			PRP + H12		
	preCPB	postCPB	post T/F	preCPB	postCPB	post T/F	preCPB	postCPB	post T/F	preCPB	postCPB	post T/F
**ELISA**												
Fibrinogen (mg/dL)	623 ± 322	193 ± 59	301 ± 54	701 ± 218	124 ± 46	284 ± 48	869 ± 391	224 ± 103	368.0 ± 102	909 ± 371	295 ± 99	330 ± 99
β-TG (ng/mL)	141 ± 22	110 ± 2	131 ± 18	135 ± 23	120 ± 13	117 ± 9	118 ± 135	112 ± 9	99 ± 11	110 ± 10	141 ± 22	82 ± 10
PF4 (ng/mL)	1.8 ± 0.3	3.9 ± 1.0	3.2 ± 1.5	2.8 ± 0.5	5.2 ± 1.3	4.3 ± 1.9	2.0 ± 0.5	4.5 ± 0.8	3.0 ± 0.6	2.4 ± 1.3	4.3 ± 1.4	2.9 ± 1.2
IL-6 (ng/mL)	0 ± 0	54.0 ± 5.4	108 ± 27.4	0 ± 0	99.8 ± 53.8	104.8 ± 67.0	0 ± 0	76.8 ± 44.9	92.1 ± 62.7	0 ± 0	53.6 ± 11.1	69.8 ± 15.9

### Involvement of H12-ADP-liposomes in thrombus formation at the bleeding site

**Figure 4:**
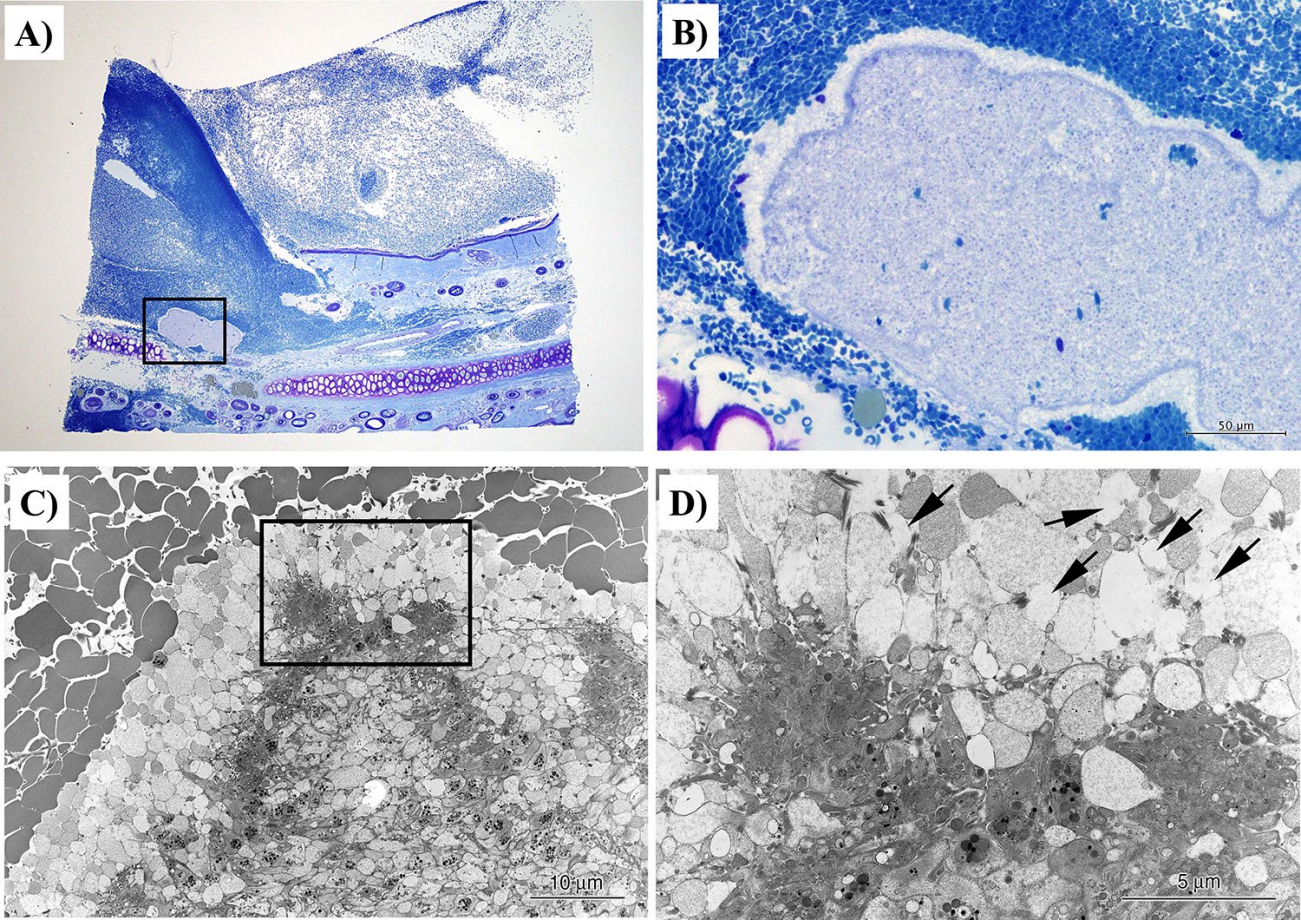
Histological studies of thrombus formation at a bleeding site in subject rabbits after H12-ADP-liposome administration following PRP transfusion. (**A**,**B**) Light micrographs of toluidine blue-stained specimens of ear bleeding sites. Platelet thrombus formation is observed in contact with the disrupted subcutaneous tissue. (**C**,**D**) Magnified images of the same part observed under scanning electron microscopy. Elliptical structures, several hundred nanometers in diameter, are observed surrounded by platelets, representing H12-ADP-liposomes (arrows) involved in thrombus formation.

We then explored the role of H12-ADP-liposome in thrombus formation at the bleeding site. Histological studies were performed under electron microscopy. We examined the auricle area where an incision was made to measure the bleeding time after H12-ADP-liposome administration following PRP transfusion in the H12-ADP-liposome/PRP group. In the vicinity of the disruption in the subcutaneous tissue, formation of platelet thrombus was observed, covered by thrombus comprising mainly erythrocytes (Fig. 4A,B). Electron microscopy of this platelet thrombus revealed several elliptical structures, several hundred nanometres in diameter, surrounded by accumulated platelets (Fig. 4C,D, arrows). Based on our previous study^14^, these structures were considered to represent H12-ADP-liposomes. These results suggested that H12-ADP-liposomes were involved in the formation of platelet thrombus.

### Effects of H12-ADP-liposome administration on the liver, lungs, and spleen

**Figure 5:**
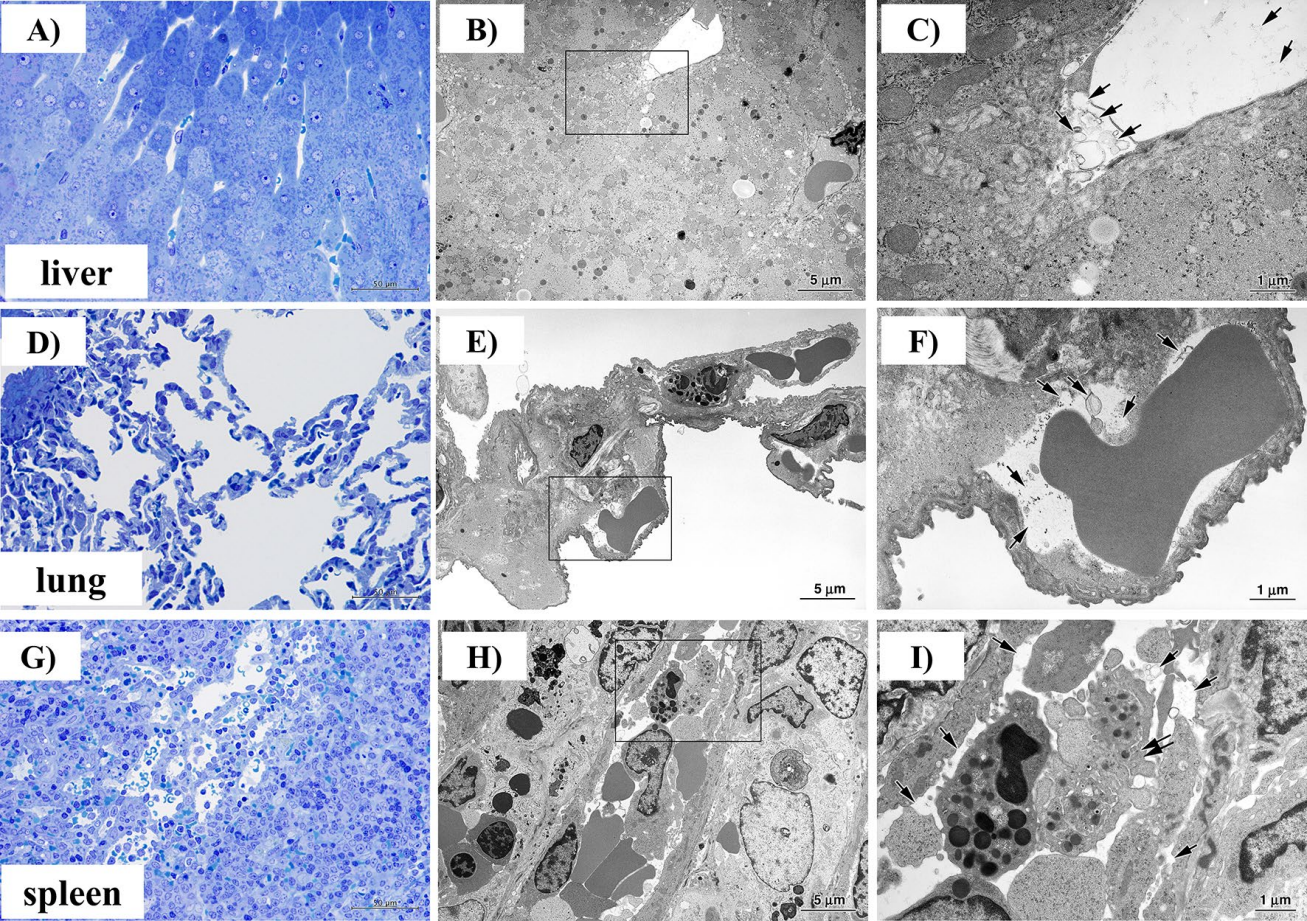
Histological studies of liver, lungs, and spleen in subject rabbits after H12-ADP-liposome administration following PRP transfusion. (**A**,**D**,**G**) Light micrographs of toluidine blue-stained specimens. (**B**,**E**,**H**) Scanning electron microscopic images of the same parts. (**C**,**F**,**I**) Magnified images of the same parts. Note that elliptical structures representing H12-ADP-liposomes are not bound to platelets and are present in blood vessels. Arrows indicate H12-ADP-liposomes. Double arrows indicate platelets.

We examined the behaviour of H12-ADP-liposomes in the peripheral blood flow of the liver, lungs, and spleen using light micrography and electron microscopy (Fig. 5A–I). In specimens from the H12-ADP-liposome/PRP group, elliptical structures considered H12-ADP-liposomes were independently present regardless of the presence of platelets in these organs (Fig. 5C,F,I). These results seemed to provide a basis for indirectly showing that thrombosis is not caused systemically by H12-ADP-liposome administration in the state of post-CPB coagulopathy.

## Discussion

In this study, administration of H12-ADP-liposomes following PRP transfusions significantly reduced bleeding time after CPB in comparison with PRP transfusion alone. H12-ADP-liposomes could act to effectively consolidate platelet haemostasis in post-CPB coagulopathy. H12-ADP-liposomes provide several advantageous features in terms of clinical applications^13,15^. First, the liposomes comprise only substances already present in the human body and have a half-life of several hours, undergoing rapid metabolism and excretion by the reticuloendothelial system. The liposomes can be stored for a long time at room temperature^24^. This represents another factor in improving convenience. H12-ADP-liposomes bind in a highly selective manner to GPIIb/IIIa receptors that are only expressed on activated platelets (not expressed on normal platelets) and are thus expected to work only at sites of vascular injury where activated platelets are located^25^ and not to cause systemic thrombosis^16,17^. Since H12-ADP-liposomes exert their haemostatic ability by aggregating activated platelets to form a thrombus, the existence of platelets that maintain their function is indispensable, and the liposomes could not work as desired under conditions of platelets that have almost lost function in terms of adhesion and aggregation responses^13^.

Cardiovascular surgery with CPB is routinely performed on a daily basis around the world; that is, post-CPB coagulopathy represents a constant concern. Fortunately, opportunities to encounter massive postoperative bleeding are decreasing, thanks to improvements in product functions such as surface coating of CPB circuits, improvements in temperature management during surgery, and shortened duration of CPB resulting from improved surgical techniques^22^. However, platelet transfusions are still required in many cases. If H12-ADP-liposomes prove feasible and effective for post-CPB coagulopathy, the effect may be significant.

Platelet dysfunction in post-CPB coagulopathy occurs when platelets come into contact with the CPB circuit or oxygenator, leading to degranulation and loss of surface receptors due to down-regulation^19,26,27^. In addition, as new intact platelets are supplied to the circulation from the bone marrow and spleen, platelets after CPB become a mixture of functioning and nonfunctioning platelets^19^. In this study, treatment with H12-ADP-liposomes and PPP failed to effectively reduce bleeding time after CPB. This may be due to the fact that H12-ADP-liposomes do not aggregate by themselves, but merely promote platelet aggregation. As demonstrated from the results of platelet function testing, almost all platelets in subject rabbits are inactivated after CPB. However, in species other than rabbits, such as in human clinical applications, a greater number of new (intact) platelets will be supplied from the bone marrow or spleen after CPB^28–30^, and some possibility remains that administration of H12-ADP-liposomes alone may prove effective.

The amount of haemorrhage after cardiovascular surgery is often evaluated as the drainage volume^31,32^, but this was difficult to measure in the present experimental CPB model due to the limited follow-up time; therefore, it could not be evaluated. However, the finding that H12-ADP-liposome administration proved effective in reducing the bleeding time suggests that the volume of clinically required platelet transfusion was suppressed, thanks to the increasing haemostatic effect and resulting reduction in haemorrhage from cardiovascular surgery using CPB. If postoperative blood transfusion can be avoided, or at least reduced by H12-ADP-liposome treatment, mortality after cardiovascular surgery and medical costs will reduce and are expected to prove very favourable. In conclusion, we believe that H12-ADP-liposomes could be an important therapeutic option for post-CPB coagulopathy and have the potential to reduce blood transfusion requirements after cardiovascular surgery with CPB.

## Methods

### Fabrication of H12-ADP-liposomes

Cholesterol and 1,2-dipalmitoyl-sn-glycero-3-phosphatidylcholine (DPPC) were obtained from Tokyo Chemical Industry (Tokyo, Japan), 1,2-distearoyl-sn-glycero-3-phosphatidylethanolamine-*N*-(monomethoxypoly[ethylene glycol]) (PEG-DSPE; 5.1 kDa) was obtained from NOF Corporation (Tokyo, Japan), 1,5-dihexadecyl-*N*-succinyl-l-glutamine (DHSG) was obtained from Nippon Fine Chemical (Osaka, Japan), and ADP was obtained from Sigma Aldrich (St Louis, MO). We synthesised H12-PEG-Glu2C18, where fibrinogen γ-chain dodecapeptide (C-HHLGGAKQAGDV [Cys-H12]) was conjugated to the end of the PEG-lipids, as described elsewhere^33^.

Mixed lipids (DPPC/cholesterol/DHSG/PEG-DSPE/H12-PEG-Glu2C18 = 5/5/1/0.033/0.033 (molar ratio)) were dissolved in ethanol (300 mg/mL), and ADP was dissolved in phosphate-buffered saline (PBS) (2.14 mg/mL). Then, 10 mL of the lipid solution was vigorously injected into 23.3 mL of ADP solution, and the mixed solution was stirred for 15 min at room temperature to prepare the liposomes. The liposomes were then extruded at room temperature to control their size (final membrane pore size: Φ0.10 µm) (LIPEX Extruder, Transferra, Canada), and the ethanol and the remaining ADP out of liposomes were removed with a crossflow system (Vivaflow 200 Laboratory Cross Flow Cassette, Sartoius, Germany). After lysing liposomes with n-octyl-β-d-glucoside (Carbosynth, UK), the concentrations of DPPC and H12-PEG-Glu2C18 were evaluated using a phospholipid measurement kit (Phospholipids C, FUJIFILM Wako Pure Chemical Corporation, Japan) and fluorescamine which reacts with H12 peptides to emit fluorescence at 495 nm, respectively. The liposome composition was determined using nuclear magnetic resonance (NMR) (INOVA400, Varian, CA, USA), and the total lipid concentration was calculated from the result of the NMR and the concentration of DPPC. Besides, H12-ADP-liposomes were dispersed into PBS and were characterised in terms of the size and the zeta potential using a Zeta sizer (Nano-ZS90, Malvern, UK). In addition, to determine the concentration of ADP inside and outside of liposomes, H12-ADP-liposomes underwent gel column chromatography (PD-10, prepacked column, GE Healthcare Japan, Japan) and were separated into fractions containing liposomes and those without liposomes. Each fraction was added to n-octyl-β-d-glucoside, and the concentration of DPPC in each fraction was determined as described above. The concentration of ADP in each fraction were also evaluated using a high-performance liquid chromatography (separation module: Water 2,695; absorbance detector: Water 2,487 (Waters Corporation, MA, USA); column: TSKgel ODS-100 V 5 µm, 4.6 mm I.D. × 25 cm (Tosoh Corporation, Japan)). Finally, the endotoxin concentration in the liposome solution was evaluated using toxinometer (ET-2000, FUJIFILM Wako Pure Chemical Corporation, Japan).

### Preparation of experimental animals

This study used male New Zealand white rabbits (body weight range, 2.8–3.3 kg; Japan SLC, Hamamatsu, Japan). All experimental procedures were approved by the institutional review board for the care of animal subjects at the National Defense Medical College (ethics approval number 17002). The investigation conforms to the guidelines from the NIH Guide for the Care and Use of Laboratory Animals. A total of 52 rabbits were used in this study and distributed as follows: 26 rabbits for the CPB model and 26 rabbits for blood donation.

### Rabbit CPB model

**Figure 6:**
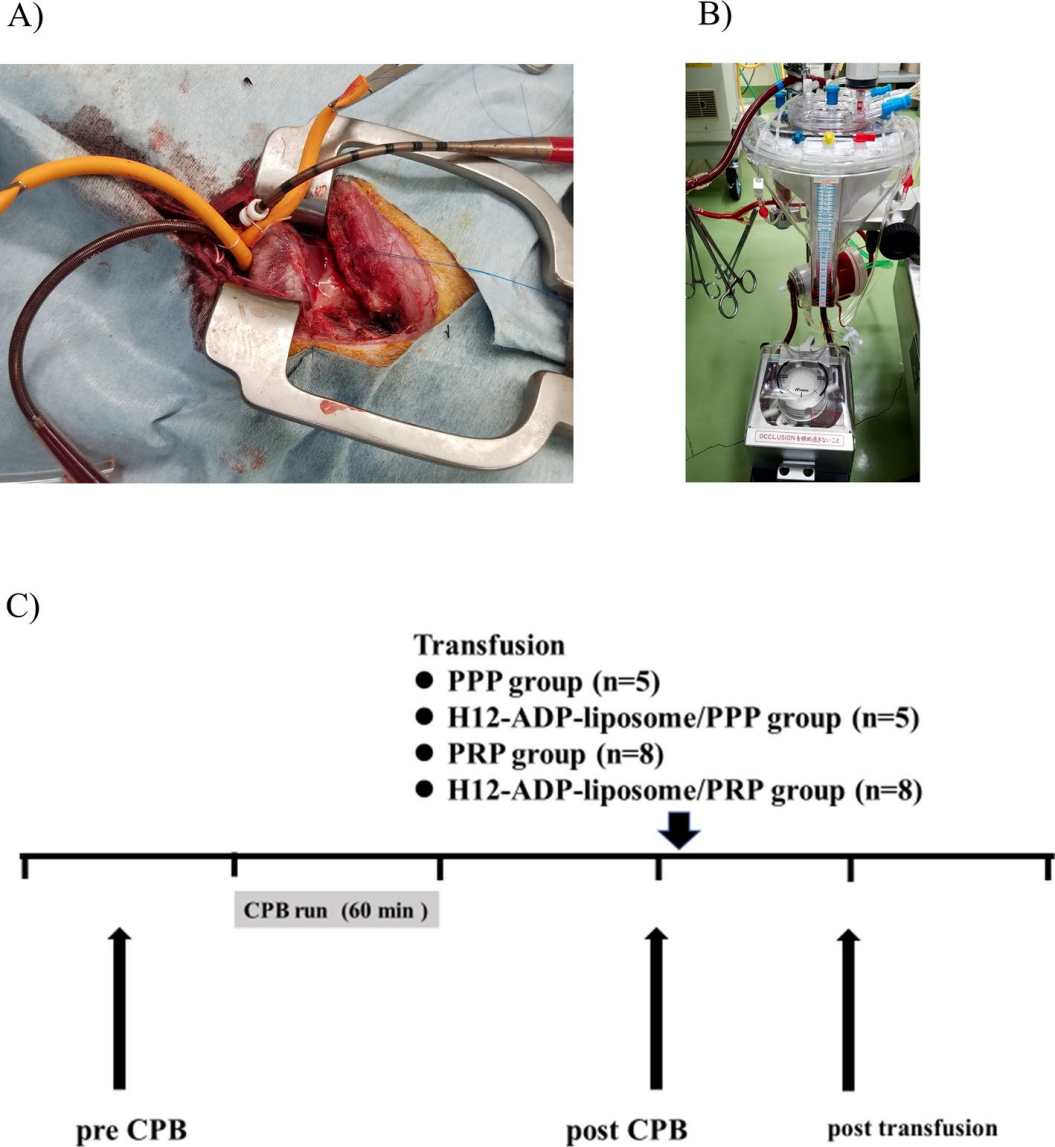
A rabbit model of cardiopulmonary bypass coagulopathy. (**A**) Cannulas in the ascending artery and right appendage are connected to the tube. (**B**) The cardiopulmonary bypass machine. (**C**) Experimental design of the current study. CPB, cardiopulmonary bypass; PPP, platelet-poor plasma; PRP, platelet-rich plasma.

All surgical procedures in this study were performed under general anesthesia and continuous monitoring of heart rate, 3-lead electrocardiography, arterial blood pressure, and oxygen saturation. After inducing anaesthesia with intramuscular ketamine (25 mg/kg) and xylazine (10 mg/kg), followed by intravenous injection of pentobarbital (15 mg/kg), animals underwent tracheotomy, followed by endotracheal intubation, and were connected to a mechanical ventilator. General anaesthesia was maintained with continuous inhalation of isoflurane (2–3%) during the procedure. The right femoral artery and vein were exposed and cannulated with a 20-gauge catheter for monitoring blood pressure and as a route of drug infusion, respectively. The CPB circuit composed of polyvinyl chloride tubing, a venous reservoir, and an oxygenator (Excelung Kids, HPO-06RHF-CP; MERA, Tokyo, Japan) was driven by a roller head pump (MERA) (Fig. 6B). The priming solution of the circuit consists of 50 mL of 25% human albumin solution (Albuminar; CSL Behring, Tokyo, Japan), 40 mL of washed red blood cells (RBCs) collected from a donor rabbit, 0.5 mL (500 IU) of heparin sodium injection, and 159.5 mL of normal saline (total volume, 250 mL). Thereafter, median sternotomy was performed and the pericardium was opened. After injecting 0.5 mL (500 IU) of heparin sodium, an 8-Fr arterial cannula was introduced into the ascending aorta and a 10-Fr venous cannula was placed in the right atrium through the right atrial appendage (Fig. 6A). These cannulas were connected to the CPB circuit and a normothermic CPB run was maintained for 60 min at a flow rate of 80–100 mL/kg/min (Fig. 6C). After weaning and termination of the CPB run, protamine sulphate (10 mg) was administered and the cannulas were removed. RBCs remaining in the CPB circuit were collected and re-administered to ameliorate severe anaemia and ensure survival.

### Preparation of washed RBCs, PRP, and PPP

Washed RBCs, PRP, and PPP were prepared in the same way, as described in detail in our previous paper^17^. After induction of general anaesthesia in rabbits in the same manner as in the CPB model, the right femoral artery was cannulated using a 20-gauge catheter. Whole blood samples were withdrawn from the catheter and collected with a 10% volume of 3.8% (w/v) sodium citrate, followed by centrifugation at 100×*g* for 15 min. The resulting supernatant was used as PRP. The remaining sample was further centrifuged at 500×*g* for 10 min, and the resulting supernatant was used as PPP. In addition, the remaining sample was collected in the same amount of ACD-A solution (Terumo Co., Tokyo, Japan) and centrifuged at 500×*g* for 10 min. The supernatant was discarded, and the remaining cells were stored in the same amount of MAP solution (Terumo Co.) and then used as washed RBC solution.

### Administration of H12-ADP-liposomes, PRP, and PPP

One hour after CPB (2 h after starting CPB), subject rabbits were randomly divided into four groups and received the following administrations: (1) 30 mL of PPP alone (PPP group; n = 5), (2) 30 mL of PPP followed by 20 mg/kg of H12-ADP-liposomes (H12-ADP-liposome/PPP group; n = 5), (3) 30 mL of PRP alone (PRP group; n = 8), and (4) 30 mL of PRP, followed by 20 mg/kg of H12-ADP-liposomes (H12-ADP-liposome/PRP group; n = 8) (Fig. 6C). H12-ADP-liposomes were administered with PBS adjusted to 20 mg/mL in solvent.

### Evaluation of complete blood count (CBC), coagulation activity, and platelet function

Whole blood samples were obtained from the femoral artery and analysed at the following three time points: (1) before surgery and CPB run (pre-CPB); (2) 1 h after finishing CPB and before administration of H12-ADP-liposomes, PPP, and/or PRP (post-CPB); and (3) 1 h after administration of H12-ADP-liposomes, PPP, and/or PRP (post-transfusion) (Fig. 1C). CBC was analysed using a haematology analyser (PEC170; Erma Inc., Tokyo, Japan). WBC count, Hb concentration, and platelet count were measured.

Coagulation activity was evaluated using a Sonoclot coagulation analyser (model SCP2; Sienco, Morrison, CO)^17^. A small amount of whole blood (400 µL) without anticoagulant was placed into a cuvette containing a glass-bead activator and a stir bar, in which a vertically vibrating probe was suspended^34^. As the sample clotted, increasing impedance to the vibration of the probe was detected by the sensor and converted to an output signal. The time of onset that fibrin formation begun was recorded as the ACT^35^.

As platelet function testing, multiple electrode impedance platelet aggregometry using a Multiplate analyser (Roche Diagnostics, Mannheim, Germany)^36,37^ was employed and collagen-induced platelet aggregation was assessed (collagen test). Whole blood samples (3 mL) were drawn into hirudin blood collection tubes (Roche Diagnostics, Mannheim, Germany) and kept at 37 °C for 30 min. Blood and reagents were pipetted using an electric pipette into test cells containing two independent sensor units. Each test was performed with 600 µL of sample blood pipetted into the test cell and stirred with a stirrer. After incubation for 3 min at 37 °C, collagen reagent (Chrono-par collagen; Chrono-log Corp, Havertown, PA) was added at a concentration of 16 µg/mL (10 µg of collagen). The adhesion and aggregation of platelets was measured by the change in electrical resistance between two sensors for 6 min. The impedance change was plotted against time, and the AUC value was recorded.

### Measurement of ear bleeding time

Ear bleeding time was measured to evaluate overall haemostatic capacity, as described below. The auricle was cut with a No. 11 surgical blade (Feather Co., Osaka, Japan) to make a 5-mm wide stab wound where no vessel was visible, and the ear was immersed in a normal saline bath at room temperature^17^. The time required for spontaneous cessation of visible bleeding was measured up to 1,200 s (20 min). Bleeding times longer than 1,200 s were recorded as 1,200 s.

### Enzyme-linked immunosorbent assay (ELISA)

Plasma samples were collected at the indicated time points and stored at − 80 °C until ELISA measurement. A commercially available rabbit fibrinogen ELISA kit (LS-F10457; LSBio, Seattle, WA), rabbit β-TG ELISA kit (MBS704354; MyBioSource, San Diego), rabbit PF4 ELISA kit (LS-F4542; LSBio, Seattle, WA), and rabbit IL-6 DuoSet ELISA (DY7984; R&D Systems, Minneapolis, MN) were used, and measurements were carried out according to the manual from the manufacturer.

### Histologic and electron microscopic study

At the end of the study, after humane euthanasia, specimens were harvested from the lungs, liver, spleen, and the part of the ear that had been incised for the measurement of bleeding time. These specimens were prefixed with fixative containing 4% paraformaldehyde and 0.5% glutaraldehyde in 0.1 mol/L phosphate buffer (pH 7.4) for 3 h at 4 °C, followed by postfixing in 1% osmium tetroxide in 0.1 mol /L phosphate buffer (pH 7.4) for 2 h at 4 °C, dehydration, and embedding in epoxy resin. For selection of the bleeding site lesion, semi-thin sections were stained with toluidine blue. Ultrathin sections stained with uranyl acetate and lead citrate were then examined under electron microscopy (JEM 1030; JEOL, Tokyo, Japan) at an accelerating voltage of 80 kV^14^.

### Statistical analysis

GraphPad Prism (version 7.04; GraphPad Software, San Diego, CA) was used for all statistical analyses. All values are expressed as mean ± standard deviation of the mean. Comparisons among groups at different time points were made by two-way analysis of variance followed by Sidak’s multiple comparisons test. Values of p < 0.05 were considered statistically significant.

## Supplementary information

Supplementary information.
