# Supplementary material for: Therapeutic potential of fibrinogen γ-chain peptide-coated, ADP-encapsulated liposomes as a haemostatic adjuvant for post-cardiopulmonary bypass coagulopathy
Source: Sci Rep. 2020 Jul 9;10:11308. doi: 10.1038/s41598-020-68307-5 (PMC7347858; doi:10.1038/s41598-020-68307-5)
Supplement: Supplementary file 1 — Supplementary information. [file 41598_2020_68307_MOESM1_ESM.pdf]

Therapeutic potential of fibrinogen γ-chain peptide-coated, ADP-encapsulated liposomes as a hemostatic adjuvant for post-cardiopulmonary bypass coagulopathy

Osamu Ishida, Kohsuke Hagisawa, Nozomu Yamanaka, Koji Tsutsumi, Hidenori Suzuki, Shinji Takeoka and Manabu Kinoshita

Results of evaluation for characteristics of H12-ADP-liposome

Liposome composition (molar ratio)
DPPC/Cholesterol/DHSG/PEG-DSPE/H12-PEG-Glu2C18 = 5.000/5.066/1.157/0.022/0.042

Size (nm)	ζ potential (mV)	ADP concentration (mg/mL)		H12-PEG-Glu2C18 (mg/mL)	Endotoxin (EU/mL)
		inside of liposomes	outside of liposomes		
153±42	-10.3±1.0	0.066	0.001	0.637	<3.1

(Total lipid concentration: 20 mg/mL) (n = 3, triplicated)
